# Spontaneous Rupture of a Leiomyoma Causing Life-Threatening Intra-Abdominal Hemorrhage

**DOI:** 10.1155/2017/3701450

**Published:** 2017-01-03

**Authors:** Melissa Schwartz, Kristin Powell

**Affiliations:** ^1^Division of Gynecologic Oncology, Department of Obstetrics, Gynecology and Reproductive Sciences, Icahn School of Medicine at Mount Sinai, 9th Floor, 1176 Fifth Avenue, New York, NY 10029, USA; ^2^Department of Obstetrics & Gynecology and Women's Health, Albert Einstein College of Medicine and Montefiore Medical Center, 5th Floor, 1300 Morris Park Avenue, Bronx, NY 10461, USA

## Abstract

*Background*. Uterine fibroids are common benign tumors in women. Clinical manifestations are well known. Acute complications necessitating emergent surgical intervention are rare.* Case*. We report a case of a 53-year-old woman with a history of uterine fibroids presenting with acute-onset severe abdominal pain. Imaging indicated massive free fluid and a large partially solid uterine mass. Vitals were consistent with hypovolemic shock. Examination revealed a surgical abdomen. She underwent an emergent laparotomy and total hysterectomy. Surgery revealed 4.5 L of hemoperitoneum and a 15 cm degenerated uterine fibroid with active bleeding. Pathology was consistent with intraoperative findings. She required transfusion of numerous blood products perioperatively. Her postoperative course was uncomplicated.* Conclusion*. It is rare for a uterine fibroid to spontaneously rupture. However, prompt recognition of this severe complication is critical for expeditious, life-saving surgical management.

## 1. Introduction

Uterine fibroids are common benign tumors in women. Most women with fibroids are asymptomatic. However, when clinically apparent, women usually experience heavy or prolonged menstrual bleeding or pelvic pressure [1]. Acute complications secondary to fibroids are rare and seldom necessitate immediate surgical intervention. Such complications include thromboembolism, acute pain due to degeneration or torsion of a pedunculated fibroid, acute urinary retention and subsequent renal failure, and acute intra-abdominal blood loss [2]. It is imperative to recognize and diagnose acute complications from fibroids as failure to do so can result in significant morbidity and even mortality. We report herein a case of spontaneous rupture of a degenerated fibroid causing life-threatening blood loss necessitating massive transfusion and emergent surgery.

## 2. Case

**Figure 1:**
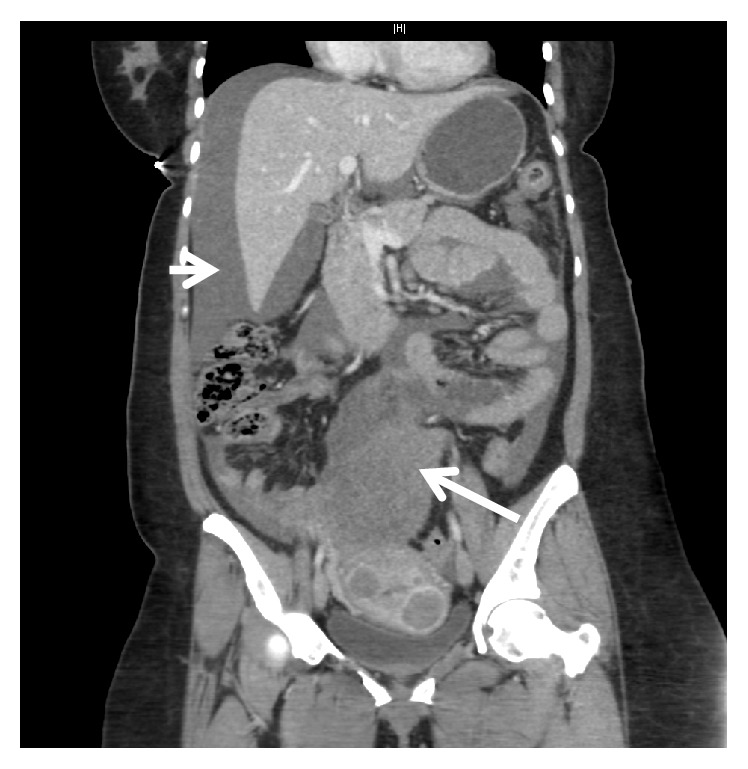
Computed tomography of the abdomen and pelvis showing the 8.8 × 7.3 × 8.3 cm degenerated fibroid (arrow) and free fluid in Morison's pouch (arrowhead).

A 53-year-old (gravida 2, para 2) perimenopausal African-American woman presented to the emergency department with worsening severe, acute-onset abdominal pain. The patient also reported dizziness and nausea. Her past medical history was significant for hypertension and known uterine fibroids. Vitals were notable for blood pressure 78/50 mm Hg and pulse rate 116 bpm. Examination revealed a 14-week-sized fibroid uterus, a distended abdomen, and diffuse abdominal tenderness with positive rebound and guarding. Hemoglobin concentration was initially 10.6 g/dL. A bedside abdominal sonogram was performed which revealed a large amount of free fluid, extending into Morison's pouch. Computed tomography, obtained by the emergency department prior to consultation, revealed an enlarged uterus with multiple fibroids as well as an 8.8 × 7.3 × 8.3 cm multicystic, partially solid, enhancing structure and moderate ascites (Figure 1). Repeat hemoglobin 2.5 hours later was 7.1 g/dL. Transfusion of 2 units of packed red blood cells was initiated. A ruptured degenerated fibroid was suspected given the patient's known history of fibroids and perimenopausal state, imaging consistent with a degenerating fibroid, and free fluid in the abdomen. However, other gynecologic sources of intra-abdominal bleeding could not be ruled out.

**Figure 2:**
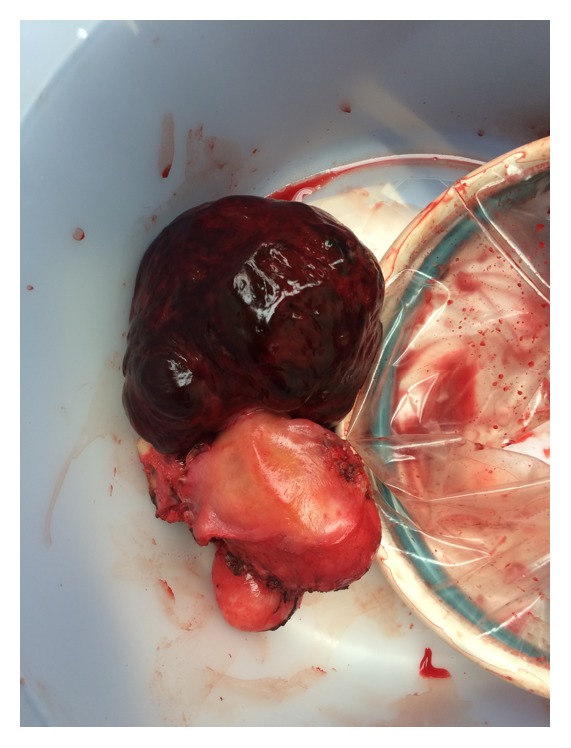
Photograph of the specimen removed. Grossly, approximately 15 cm exophytic red degenerated fibroid.

Given the acute nature of the patient's intra-abdominal bleeding and deteriorating hemodynamic status, she was taken emergently to the operating room for exploratory laparotomy. Prior to induction of anesthesia, the patient's blood pressure nadired at 60/53 and pulse was in the 140's. Vasopressors were started and additional transfusion products were given. Upon entry into the abdomen, approximately 4.5 liters of hemoperitoneum was observed. Intraoperative findings were significant for a ruptured degenerated fundal subserosal fibroid, which was actively bleeding posteriorly (Figure 2). The patient's uterus was fibromatous. Given the severity of the bleeding, the decision was made to perform a total hysterectomy. Additional blood loss from the case was 500 milliliters. In total, she received 6 units of packed red blood cells, 3 units of fresh frozen plasma, 1 unit of platelets, and 5 liters of crystalloid. The patient's postoperative course was uneventful and she was discharged home three days after surgery. Histopathologic review of the specimen revealed multiple fibroids, the largest being 15 cm with extensive ischemic hemorrhagic and hyalinizing degenerative changes.

## 3. Discussion

Although uterine fibroids are very common, they infrequently cause acute complications. The spontaneous rupture of a degenerated fibroid is extremely rare with only around 10 cases reported in the last half decade [3, 4]. Significant bleeding from a ruptured fibroid is even more unusual [5, 6]. This case documents rupture of a degenerated fibroid causing acute hemorrhage requiring a massive blood transfusion.

Intraperitoneal hemorrhage from rupture of a fibroid is usually venous and secondary to an increase in abdominal pressure, which causes rupture of superficial veins. Less often, bleeding can be arterial and associated with hypertension. Trauma causing avulsion of a fibroid, torsion of a pedunculated fibroid, and pregnancy causing venous congestion resulting in vessel rupture are other possible etiologies for intra-abdominal bleeding from a fibroid [2, 4, 6–8]. In our case, the most likely cause was from degeneration of a pedunculated fibroid. This resulted in necrosis and spontaneous perforation of the fibroid's posterior surface and bleeding into the abdomen. Hyaline and red degeneration were noted on pathologic examination of the specimen removed. Hyaline degeneration is the most common degenerative change (63% of cases), whereas red degenerations account for only ~3% of uterine fibroid degenerations. Fibroid degeneration results from excessive growth that exceeds the blood supply or from mechanical compression of feeder vessels [9].

Although the pathogenesis and degeneration of fibroids are incompletely understood, steroid hormones are known to play a role [10]. Fibroids are responsive to estrogen and progesterone, with symptomatic fibroids being uncommon in prepubertal and postmenopausal women [11]. Interestingly, both pregnancy and menopause have been associated with a decrease in size and risk of fibroid formation [12–14]. Although pregnancy causes an increase in estrogen levels and menopause decreases estrogen levels, both conditions are associated with a lack of menstrual cyclicity. Therefore, menstrual cyclicity has also been implicated to be important to maintenance and growth of fibroids. As the patient in this case was perimenopausal, one mechanism for degeneration of her fibroid could be directly related to decreasing levels of estrogen and progesterone in combination with menstrual cyclicity irregularity. Hormonal changes likely affected angiogenesis and vascular blood supply to her large, dominant fibroid, ultimately causing red degeneration.

Imaging modalities can aid in diagnosis of hemoperitoneum. However, computed tomography and ultrasound may not be able to delineate the origin of the bleeding [15]. Timely diagnosis and rapid emergent surgical intervention can be life-saving in situations such as the case described. Even though acute hemorrhage from a degenerated fibroid is rare, it should be included on the differential in any women with an acute intra-abdominal bleed and a history of fibroids.

## Additional Points

- Spontaneous rupture of a uterine fibroid is rare.
- Ruptured degenerated fibroid should be included on the differential in the situation of an acute abdomen and massive hemoperitoneum.
- Prompt diagnosis and management of a ruptured fibroid can be life-saving.
